# Fine-Tuning of *Arabidopsis thaliana* Response to Endophytic Colonization by *Gluconacetobacter diazotrophicus* PAL5 Revealed by Transcriptomic Analysis

**DOI:** 10.3390/plants13131719

**Published:** 2024-06-21

**Authors:** Fabiano Silva Soares, Ana Lídia Soares Rangel de Souza, Suzane Ariádina de Souza, Luciano de Souza Vespoli, Vitor Batista Pinto, Lucia Matiello, Felipe Rodrigues da Silva, Marcelo Menossi, Gonçalo Apolinário de Souza Filho

**Affiliations:** 1 Laboratório de Biotecnologia (Unidade de Biologia Integrativa), Universidade Estadual do Norte Fluminense Darcy Ribeiro (UENF), Campos dos Goytacazes, Rio de Janeiro 28013-602, Brazil; soares_fabiano@hotmail.com (F.S.S.); analidiarangel@yahoo.com.br (A.L.S.R.d.S.); suzaneariadina@hotmail.com (S.A.d.S.); lucianovespoli@gmail.com (L.d.S.V.); 2 Laboratório de Biologia Celular e Tecidual, UENF, Campos dos Goytacazes, Rio de Janeiro 28013-602, Brazil; vitorbp@uenf.br; 3 Instituto de Biologia, Departamento de Genética, Evolução, Microbiologia e Imunologia, Universidade Estadual de Campinas (UNICAMP), Campinas, São Paulo 13083-970, Brazil; lucia@lgf.ib.unicamp.br (L.M.); felipe.silva@embrapa.br (F.R.d.S.); menossi@lgf.ib.unicamp.br (M.M.); 4 Embrapa Agricultura Digital, Campinas, São Paulo 13083-886, Brazil

**Keywords:** RNA-seq, beneficial endophyte, growth promotion, plant defense

## Abstract

*Gluconacetobacter diazotrophicus* is a diazotrophic endophytic bacterium that promotes the growth and development of several plant species. However, the molecular mechanisms activated during plant response to this bacterium remain unclear. Here, we used the RNA-seq approach to understand better the effect of *G. diazotrophicus* PAL5 on the transcriptome of shoot and root tissues of *Arabidopsis thaliana*. *G. diazotrophicus* colonized *A. thaliana* roots and promoted growth, increasing leaf area and biomass. The transcriptomic analysis revealed several differentially expressed genes (DEGs) between inoculated and non-inoculated plants in the shoot and root tissues. A higher number of DEGs were up-regulated in roots compared to shoots. Genes up-regulated in both shoot and root tissues were associated with nitrogen metabolism, production of glucosinolates and flavonoids, receptor kinases, and transcription factors. In contrast, the main groups of down-regulated genes were associated with pathogenesis-related proteins and heat-shock proteins in both shoot and root tissues. Genes encoding enzymes involved in cell wall biogenesis and modification were down-regulated in shoots and up-regulated in roots. In contrast, genes associated with ROS detoxification were up-regulated in shoots and down-regulated in roots. These results highlight the fine-tuning of the transcriptional regulation of *A. thaliana* in response to colonization by *G. diazotrophicus* PAL5.

## 1. Introduction

The increase in human population has resulted in the growing demand for agricultural production. As a result, the use of industrialized fertilizers has significantly expanded in recent decades. Plant-growth-promoting bacteria (PGPB) have emerged as a suitable alternative to counteract the adverse environmental impacts exerted by synthetic agrochemicals [1]. In addition to the effect on plant growth and development, the colonization by PGPB may activate plant defense, protecting plants against a wide range of pathogens and abiotic factors [2,3]. PGPB can be used as biofertilizers and biocontrol agents, promoting plant growth and health.

*Gluconacetobacter diazotrophicus* is an endophytic and diazotrophic PGPB, first isolated from sugarcane [4]. This bacterium can live inside the tissues of several plant species of agronomic relevance, improving their growth and productivity [5]. The ability of *G. diazotrophicus* to stimulate plant growth results from various mechanisms, including nitrogen biological fixation, production of phytohormones, solubilization of mineral nutrients, and inhibition of phytopathogens [6,7]. In 2009, the genome of the PAL5 strain of *G. diazotrophicus* was fully sequenced, and the genes involved in nitrogen biological fixation, sugar metabolism, transport systems, polysaccharides biosynthesis, quorum sensing, and auxin biosynthesis were identified [8].

Over the last decades, several studies have addressed the identifying genes and regulatory proteins involved in the interaction of *G. diazotrophicus* PAL5 with host plant species, particularly in monocots such as sugarcane [9,10,11,12]. However, many of the crops of agronomic interest are slow-growing species with a complex genome, and this often hinders their cultivation under controlled conditions and limits the detailed molecular characterization of the plant–bacteria association [13]. Due to its short 6-8 week lifespan, easy maintenance, sequenced genome, small space requirements, and a genome of five chromosomes [14], *A. thaliana* is a model plant for studies of how plants and microorganisms interact [15]. Several studies have already demonstrated the usefulness of *A. thaliana* for the characterization of the plant–PGPB association [16,17,18].

In a previous study, we demonstrated the ability of *G. diazotrophicus* PAL5 to establish an endophytic association within the roots of *A. thaliana*, resulting in biomass gain and increased photosynthetic capacity and water-use efficiency 50 days after inoculation [19]. In addition, the authors showed that the plant defense system is activated during the initial stage of the association of the bacterium and the plant host [19]. Other studies have also shown that the interaction between *G. diazotrophicus*–*A. thaliana* activates plant defense mechanisms [13,20]. Thus, *A. thaliana* is useful for molecular studies of the beneficial interaction with *G. diazotrophicus* PAL5 [19]. Proteomic analysis has revealed the proteins and metabolic pathways of *G. diazotrophicus* PAL5 activated during its co-cultivation with *A. thaliana* [21]. However, the molecular mechanisms regulated in *A. thaliana* plants as a response to endophytic colonization by *G. diazotrophicus* PAL5 remain poorly understood.

With the advancements of omics technologies, the knowledge about the complex molecular interactions between plants and PGPB has been improved. The elucidation of such mechanisms may optimize PGPB use on different crops and environmental conditions, as well as the formulation of bio-based commercial products [22]. RNA sequencing (RNA-seq) has been applied as a powerful tool for transcriptome analysis, allowing the characterization of the plant response to PGPB applications [23,24].

The present study has investigated the molecular mechanisms activated during the growth response of *A. thaliana* to colonization by *G. diazotrophicus* PAL5 in shoot and root tissues. Plants were inoculated with *G. diazotrophicus* PAL5 and, after plant growth confirmation, the gene expression was analyzed at 50 days post-inoculation, using transcriptomic analysis, via RNA-seq. The main plant genes and mechanisms regulated during the interaction with *G. diazotrophicus* PAL5 were characterized.

## 2. Results

### 2.1. Effects of Gluconacetobacter diazotrophicus PAL5 on Plant Growth and Colonization

**Figure 1:**
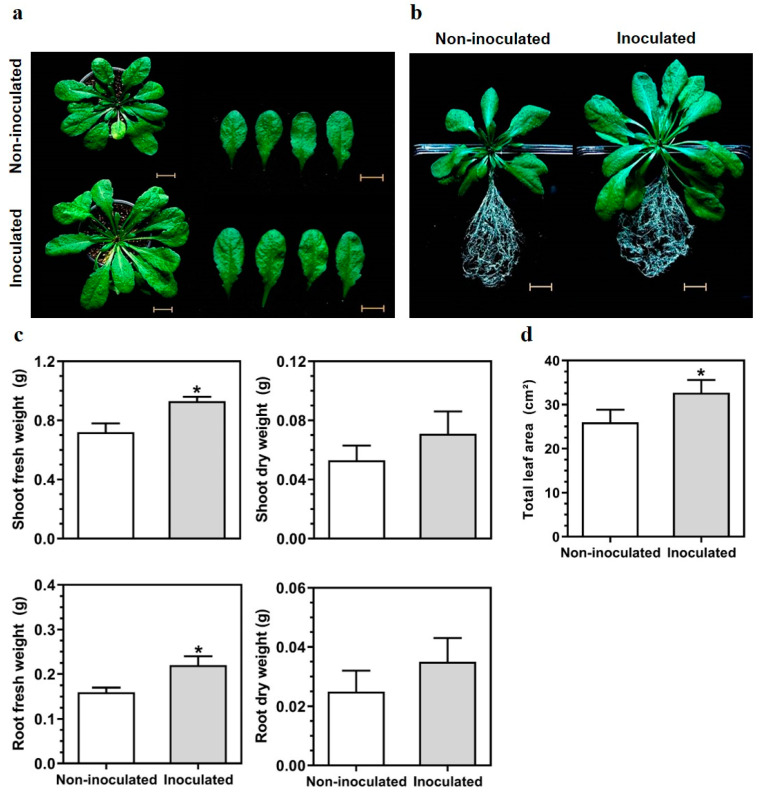
The growth-promoting effect of *Gluconacetobacter diazotrophicus* PAL5 on *Arabidopsis thaliana* at 50 dpi: (**a**) Morphological effect on rosette growth; (**b**) Morphological effects on root growth; (**c**) The fresh and dry weights of shoots (higher) and roots (bottom); (**d**) Total leaf area. Seven-day-old seedlings were inoculated with *G. diazotrophicus* strain GD-Kan (1 × 10^6^ CFU/mL^−1^). Seedlings treated with water served as a control. The data shown represent the means of 20 biological replicates. Error bars represent the SD. “*” represents statistically significant differences between treatments (ANOVA, Tukey’s test; *p* < 0.05). Bar = 1 cm.

Aiming to investigate the effect of inoculation by *G. diazotrophicus* PAL5 on the plant growth of *A. thaliana*, ten-day-old seedlings were inoculated, and plants were cultivated for 50 days (Figure 1a,b). At 50 dpi, the inoculation of *G. diazotrophicus* PAL5 on plants increased the shoot and root fresh weight by 29.2% and 37.5%, respectively, when compared to the control plants (Figure 1c). *G. diazotrophicus* PAL5 also increased shoot and root dry weight by 34.0% and 40.0%, respectively (Figure 1c). The total leaf area increased by 25.9% when plants were inoculated with *G. diazotrophicus* PAL5 (Figure 1d).

**Figure 2:**
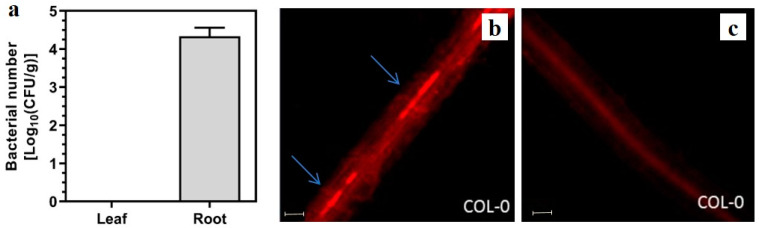
Endophytic colonization of *A. thaliana* plants by *G. diazotrophicus* PAL5 at 50 dpi: (**a**) Counting values of *G. diazotrophicus* PAL5 strain GD-Kan in the leaves and roots of *A. thaliana* plants; (**b**) *A. thaliana* roots inoculated with *G. diazotrophicus* PAL5 strain GD-F expressing the *rfg* gene; (**c**) Control (without inoculation). Bars = 2 µm.

The bacterial number of *G. diazotrophicus* PAL5 in the roots and leaves of plants was investigated by CFU analyses. The bacterial population within *A. thaliana* roots was approximately 1 × 10^5^ CFU/g, at 50 dpi (Figure 2a), but no bacteria were detected in leaf tissues (Figure 2a). Aiming to confirm the presence of the bacterium inside the root tissues, *A. thaliana* was inoculated with a DsRed Fluorescent Protein-labeled *G. diazotrophicus* PAL5 strain, and an epifluorescence microscopy technique was applied. The results revealed the bacteria associated with the xylem lumen of the roots of inoculated plants (Figure 2b). The control plants without inoculated bacteria showed no fluorescent cells (Figure 2c).

### 2.2. Transcriptomic Changes Induced by Gluconacetobacter diazotrophicus PAL5 in the Shoots and Roots of Arabidopsis thaliana

The transcriptomic response induced in the shoots and roots of *A. thaliana* by *G. diazotrophicus* PAL5 was analyzed by RNA-seq. A total of 12 independent libraries, 6 from shoots (SC1, SC2, and SC3 from non-inoculated controls and SI1, SI2, and SI3 from inoculated plants) and 6 from roots (RC1, RC2, and RC3 from non-inoculated controls and RI1, RI2, and RI3 from inoculated plants) were sequenced using the Illumina HiSeq platform. A total of 190.6 and 150.6 million raw reads were generated for shoots and roots in both treatments, respectively. The value of Q30% was above 90.5% for all libraries in this sequencing. Among all the libraries, the percentage of total mapped reads and multiple alignments to the *A. thaliana* reference genome in both organs was above 84.2% and 2.9%, respectively (Table 1).

### 2.3. Analysis of Differentially Expressed Genes (DEGs)

**Figure 3:**
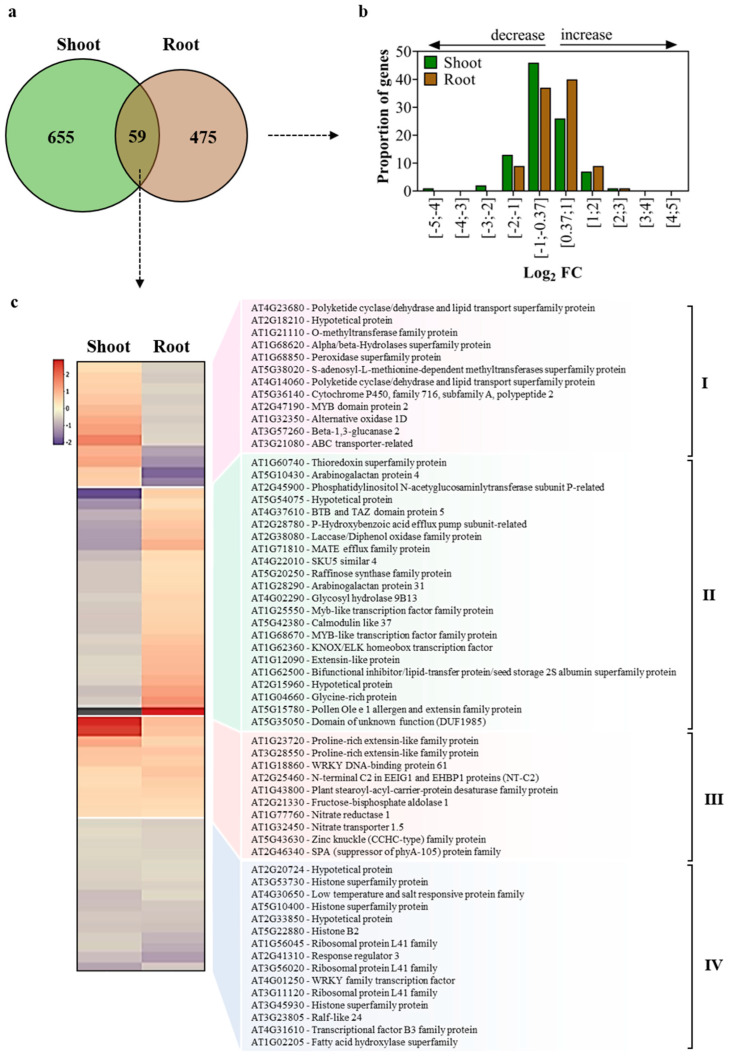
Overview of *Arabidopsis thaliana* genes differentially regulated in shoots and roots in response to colonization by *Gluconacetobacter diazotrophicus* PAL5 at 50 dpi: (**a**) Venn diagram of genes differentially regulated in response to *G. diazotrophicus*. In total, 1189 genes were differentially regulated. Overlaps show the number of genes regulated in both tissues; (**b**) Proportion of genes at different fold change (FC) ranges. The log_2_ FC intervals are positive for up-regulated genes and negative for down-regulated genes; (**c**) Heatmap of differentially expressed genes (DEGs) that were detected in both shoot and root tissues (overlapping group of Venn diagram). Up-regulated (red), down-regulated (blue).

The FPKM counts revealed a high number of DEGs in the shoots and roots from plants inoculated with *G. diazotrophicus* PAL5 (Table S1). A total of 714 and 534 DEGs were observed in shoot and root tissues, respectively. Among the genes regulated in shoots, 256 were up-regulated, and 458 were down-regulated. Among the DEGs from roots, 277 were up-regulated, and 257 were down-regulated. The density of regulated genes in each organ of the inoculated plant was analyzed according to the expression levels of the log_2_ fold change (Figure 3b). In shoots, the highest density of regulated genes was found in the range [−1 to −0.37]. In contrast, it was found in the range [0.37 to 1] in roots (Figure 3b).

The analyses revealed 655 and 475 genes regulated exclusively in shoots and roots, respectively (Figure 3a). Only 59 genes were regulated in both organs of the inoculated plants. Therefore, inoculation with *G. diazotrophicus* PAL5 generates different molecular responses among the organs of *A. thaliana*. The 59 DEGs regulated in both shoot and root tissues were grouped according to the expression pattern via heatmap analysis (Figure 3c). The results revealed that 34 of these genes had opposite regulations between organs: 12 DEGs were up-regulated in shoots and down-regulated in roots (Figure 3c, group I), and 22 DEGs were down-regulated in shoots and up-regulated in roots (Figure 3c, group II). Only 25 DEGs were similarly regulated in both plant organs, with 10 DEGs being up-regulated (Figure 3c, group III) and 15 down-regulated (Figure 3c, group IV).

### 2.4. Functional Annotation Analysis of DEGs

**Figure 4:**
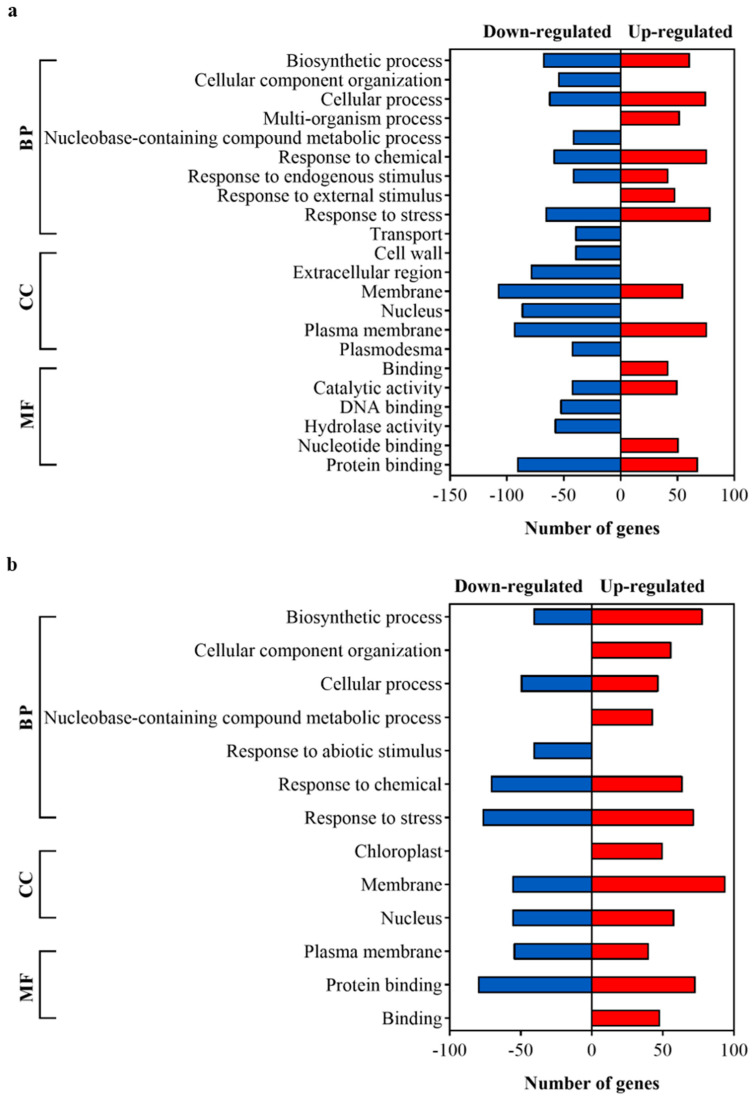
Identification of differentially expressed genes (DEGs) and its related functional classification of Gene Ontology (GO) terms in the shoot (**a**) and root (**b**) tissues of *Arabidopsis thaliana* inoculated with *Gluconacetobacter diazotrophicus* PAL5 at 50 dpi. GO terms less than 40 DEGs are shown in Supplementary Table S2. BP = biological processes; CC = cellular components; MF = molecular functions.

GO analysis was performed to obtain a functional view of the DEGs. The DEGs were grouped into three main categories—biological processes, cellular components, and molecular functions—using the OmicsBox software [25]. Figure 4 shows the most represented GO terms of the three categories. GO terms with less than 40 genes are presented in Supplementary Table S2.

In shoots (Figure 4a), in the category “biological processes—BP”, the largest group of up-regulated genes was related to “stress response”, and the main groups of down-regulated genes were related to “biosynthetic process” and “stress response”. In the category of “cellular components—CC”, the largest group of up-regulated genes was related to the “plasma membrane”, and the largest group of down-regulated genes was related to the “membrane”. In the “molecular functions—MF category”, the largest group of DEGs was related to “protein binding” for both up- and down-regulated genes.

In roots (Figure 4b), in the category “biological processes—BP”, the largest group of up-regulated genes was related to the “biosynthetic process”, and the largest group of down-regulated genes was related to “stress response”. In the “cellular components—CC” category, the largest groups of up- and down-regulated genes were related to “membrane”. In the “molecular functions—MF” category, the main groups of up- and down-regulated genes were related to “protein binding”.

### 2.5. Main Regulated Pathways in Arabidopsis thaliana in Response to the Association with Gluconacetobacter diazotrophicus PAL5

**Figure 5:**
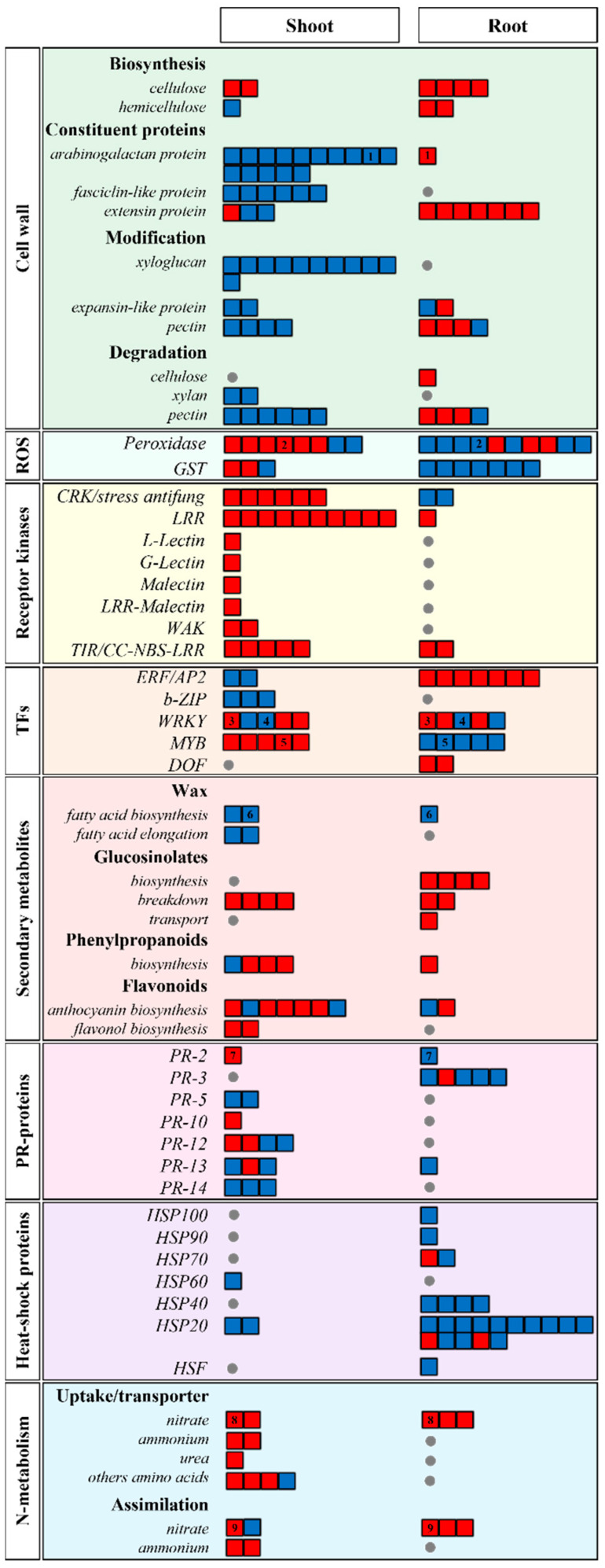
Differentially expressed genes in the shoots and roots of *Arabidopsis thaliana* plants inoculated with *Gluconacetobacter diazotrophicus* PAL5 (at 50 dpi). Each gene is represented as a box; red boxes indicate genes up-regulated, and blue boxes indicate those down-regulated. The regulation of genes is based on log_2_ FC (*p* < 0.05). Squares with the same number indicate genes regulated in both plant tissues. The chart was compiled with the Adobe Illustrator program, using the MapMan functional categories. See Table S3 for details.

To obtain a more detailed view of the main biological pathways regulated in *A. thaliana* shoots and roots in response to inoculation with *G. diazotrophicus* PAL5, the 1,189 described DEGs were organized into functional groups according to their prominent biological roles defined by MapMan [26]. Among these, 262 DEGs related to the main regulated pathways (Table S3, Figure 5) are described below:

#### 2.5.1. Cell Wall Biosynthesis/Metabolism

A group of 77 DEGs related to cell wall biosynthesis/metabolism was identified in inoculated plants (Figure 5). However, the DEGs of such pathways showed opposite regulation among shoot and root tissues. In shoots, the DEGs were mostly repressed (49 down-regulated and 3 up-regulated), while most DEGs were induced in roots (22 up-regulated and 3 down-regulated).

Many DEGs related to the biosynthesis of cell wall components, detected in shoots, are members of the arabinogalactan’s protein group (AGPs involved in differentiation, cell–cell recognition, embryogenesis, and programmed cell death). These DEGs include 15 arabinogalactan proteins (AGP1, at5g64310; AGP4, at5g1043; AGP5, at1g35230; AGP7, at5g65390; AGP9, at2g14890; AGP12, at3g13520; AGP13, at4g26320; AGP14, at5g56540; AGP17, at2g23130; AGP18, at4g37450; AGP20, at3g61640; AGP23, at3g57690; AGP24, at5g40730; AGP25, at5g18690; and AGP26, at2g47930) and six fasciclin-like proteins (FLA2, at4g12730; FLA7, at2g04780; FLA9, at1g03870; FLA11, at5g03170; FLA13, at5g44130; and FLA12, at5g60490).

In shoots, all 17 genes involved in cell wall modification were repressed, such as xyloglucan endotransglucosylase (XTH3, at3g25050; XTH4, at2g06850; XTH5, at3g23730; XTH6, at5g65730; XTR7, at4g14130; XTH8, at1g11545; XTH9, at4g03210; XTH17, at1g65310; XTH22, at5g57560; XTH30, at1g32170; and XTH33, at1g10550), pectin methyl esterase (PME8, at1g05310; PME4, at2g47030; PME5, at2g47040; and PME49, at5g07420), and expansin-like protein (EXLA2, at4g38400; and EXLA3, at3g45960). In addition, shoot tissues also showed the repression of eight genes involved with cell wall degradation (BXL1, at5g49360; BXL2, at1g02640; and at1g02790, at1g70500, at3g01270, at3g07850, at3g14040, and at5g48140).

In root tissues, genes encoding extensin proteins (at1g12040, at1g26240, at1g26250, at1g62440, at2g15880, at4g08400, and at4g08410) and pectinase (at1g09890, at1g10640, and at1g60590) were induced. In addition, four other cell–wall-degrading enzymes (at1g19940, at1g09890, at1g60590, and at1g10640) were also induced. All DEGs related to cellulose synthesis (IRX1, at4g18780; IRX3, at5g17420; IRX5, at5g44030; and IRX6, at5g15630) and the hemicellulose (at1g19300 and at5g20260) of the secondary wall were induced in roots, suggesting an activation of the remodeling and reinforcement cell wall at the site of the colonization of the bacterium.

#### 2.5.2. ROS Detoxification

The colonization by *G. diazotrophicus* PAL5 also regulated the expression of several genes of *A. thaliana* involved in ROS detoxification (Figure 5). These genes were mainly induced in shoots (8 up-regulated and 3 down-regulated) but repressed in roots (3 up-regulated and 14 down-regulated). In shoots, six peroxidase genes (PER11, at1g68850; PER20, at2g35380; PER23, at2g38390; PER27, at3g01190; PER32, at3g32980; and PER54, at5g06730) were induced. Similarly, three other peroxidase genes (PER12, at1g71695; PER16, at2g18980; and PER28, at3g03670) were induced in roots. The PER11 gene (at1g68850) was induced in shoots but repressed in roots. The genes encoding PER66 (at5g51890) and PER71 (at5g64120) were repressed in both organs of the inoculated plant. The genes encoding the glutathione S-transferase enzyme (GST) were repressed in roots. Among these genes, four genes belonged to the tau class (GSTU5, at2g29450; GSTU9, at5g62480; GSTU11, at1g69930; and GSTU25, at1g17180) and two to the phi class (GSTF6, at1gL02930; and GSTF7, at1g02920). In shoots, two GST genes were induced, both from the phi class (GSTF12, at5g17220; and GSTF14, at1g49860), while a tau class gene was repressed (GSTU17, at1g10370).

#### 2.5.3. Kinase Receptors

A total of 32 DEGs encoding kinase receptors were detected in response to inoculation, mainly on shoot tissues, where all 27 DEGs detected were induced. Only five DEGs of receptor kinases were detected in roots, three induced and two repressed (Figure 5).

Among the 27 genes induced in shoots, 22 encode receptor-like kinases (RLKs), including 10 RLK genes with the LRR domain (members of the receptor-like protein—RLP family), six members of the cysteine-rich receptor-like kinase family, two members of the wall associated-kinase (WAK) family, one RLK with the L-lectin domain, one G-lectin domain, one malectin domain, and one LRR-malectin domain. The other five DEGs induced in shoots encode TIR-NBS-LRR cytoplasmic receptors.

Among the five kinase receptors regulated in roots, one RLP (Flg22, at2g19190) and two cytoplasmic receptors CC-NBS-LR were induced. In addition, two members of the CRK family were repressed.

#### 2.5.4. Transcription Factors

The colonization by *G. diazotrophicus* PAL5 regulated 31 transcription factor (TF) genes in *A. thaliana*. In shoots, 15 DEGs encoding TFs were detected (8 up-regulated and 7 down-regulated). In roots, 19 DEGs encoding TFs were detected (12 up-regulated and 7 down-regulated) (Figure 5). Three families of TFs (ERF/AP2, WRKY, and MYB domain) were detected in both organs, and b-ZIP domain and DOF domain families were exclusively detected in shoots and roots, respectively.

In shoots, five MYB genes were induced, including MYB114 and at1g70000, involved in anthocyanin metabolism. The genes WRKY47, WRKY61, and WRKY75, which are involved in the defense response, were also induced. On the other hand, three members of the bZIP family and five members of the AP2-ERF family (APETALA2/ethylene response factor) were repressed. Among those, RAP2.6, belonging to the AP2-EREB superfamily, had a higher repression (1.513-fold).

WRKY61 and WRKY22 were both induced in shoots and repressed in roots. WRKY46 and WRKY42 were induced in roots, while WRKY30 was repressed 1.696-fold. Members of the MYB family were also repressed in roots, including MYB2 and MYB108.

Table S4 shows the TF genes regulated in the shoots and roots of *A. thaliana* plants inoculated with *G. diazotrophicus* PAL5.

#### 2.5.5. Biosynthesis/Degradation of Secondary Metabolites

Thirty-three DEGs related to the biosynthesis and degradation of secondary metabolites were found in shoots and roots (Figure 5). Among such DEGs, only 1 was regulated in both organs, while 22 DEGs were regulated exclusively in shoots (16 up-regulated and 6 down-regulated), and 10 DEGs were exclusively regulated in roots (9 up- and 1 down-regulated).

Four DEGs involved with the wax metabolism pathway were repressed in both shoot and root tissues. Notably, the CER1 gene (at1g02205) showed greater repression in the shoot. Eleven DEGs involved in the glucosinolate metabolism pathway were induced in inoculated plants. In roots, four genes participate in the biosynthesis of glucosinolates (CYP79B3, at2g22330; CYP83A1, at4g13770; BCAT4, at3g19710; and MAN1, at5g23010), two genes act on degradation (TGG1, at5g26000; and TGG2, at5g259), and one is involved in transport (BASS5, at4g12030). In shoots, only genes involved in the degradation of glucosinolates were observed (at1g54020; MBP1, at1g52040; and NIT4, at5g22300).

Five DEGs related to the biosynthetic pathway of phenylpropanoids were detected. In shoots, three DEGs were induced (UGT72E1, at3g50740; UGT72E2, at5g66690; and OMTF3, at3g61990), and one was repressed (PRR1, at1g32100). One DEG was induced in roots (CYP84A1, at4g36220).

Eleven DEGS codifying the components of flavonoid biosynthesis pathways were identified. In shoots, nine DEGs were regulated (seven for anthocyanin and two for flavonol biosynthesis). Among these, seven DEGs were induced, and only two were repressed. In roots, only two DEGs related to flavonoid biosynthesis were identified, both involved in anthocyanin biosynthesis.

#### 2.5.6. Pathogenesis-Related Proteins

Twenty DEGs encoding pathogenesis-related (PR) proteins were regulated in shoots and roots (Figure 5). The PR-2 protein gene (at3g57260) was induced in shoots but repressed in roots. The five members of the PR-3 protein family were differentially expressed only in roots (one up-regulated and four down-regulated). On the other hand, members of the PR-5 (two down-regulated), PR-10 (one up-regulated), PR-12 (two up- and two down-regulated), and PR-14 (three down-regulated) families were exclusively detected in shoots. DEGs of the PR-13 protein family were also identified in both organs of the inoculated plants: three DEGs in shoots (one up-regulated and two down-regulated) and one DEG in roots (down-regulated).

#### 2.5.7. Heat-Shock Proteins

Several DEGs encoding heat-shock proteins (HSPs) were repressed in inoculated plants, with an emphasis on root tissues (Figure 5). Among the 26 HSP genes detected in both organs, 23 were repressed, and only 3 were induced. The HSP genes belonging to the families HSP100 (at1g74310), HSP90 (at5g52640), HSP70 (at1g16030), and HSP40 (at1g71000, at1g72416, at2g20560, and at3g08970), as well to the HSF (at2g26150), were exclusively repressed in roots. In contrast, HSP60 (at3g62570) was differentially expressed only in shoots (down-regulated). The largest family of repressed HSPs belongs to the HSP20 group, including 2 in shoots (at2g17880 and at5g04890) and 12 in roots (at1g07400, at1g52560, at1g53540, at1g54050, at1g59860, at2g29500, at4g10250, at4g2520, at4g2520, at4g2520, at4g2520, at4g2520, at4g2520, at4g2520, at4g2520, and at4g62020). Among the three DEGs induced in roots are one HPS70 (at1g09080) and two HPS20 (at4g36040 and at5g47590).

#### 2.5.8. Nitrogen Metabolism and Transport

A set of 17 DEGs involved in the assimilation, uptake, and transport of nitrogen was identified, most of which were up-regulated in both organs of the inoculated plants (Figure 5). Among them, 11 DEGs involved with nitrogen uptake (nitrogen sources) and transport systems were identified in shoots and roots. The NRT1.5 gene (at1g32450), a member of the NRT1 family that encodes the low-affinity transport system (LAT), was induced in both shoot and root tissues. Shoots and roots also showed the induction of genes of the NRT2 family (high-affinity transport system—HAT), including the NRT2.1 (at1g08090) and NRT2.4 (at5g60770) genes in roots and the NRT2.5 gene (at1g12940) in shoots. In addition, in shoots, two ammonium transporter genes (AMT1-2, at1g64780 and AMT1-3, at3g24300), one urea transporter gene (DUR3, at5g45380), and three amino acid transporter genes (CAT5, at2g34960; CAT1, at4g21120; and APP3, at1g77380) were induced. The amino acid transporter gene (APP7, at5g23810) was repressed in shoots.

In the nitrogen assimilation pathway, the NIA1 gene (at1g77760), which encodes the enzyme nitrate reductase (NR), was induced in shoots and roots, and the nir1 gene (at2g15620), encoding the enzyme nitrite reductase (NiR), was induced only in roots. Two genes involved with ammonium assimilation, belonging to the GLN1 gene family (GLN1-4, at5g16570 and GLN1-1, at5g37600), were induced in shoots. Two DEGs with a regulatory role in nitrogen assimilation were identified: KING1 (at3g48530), induced in roots, and FUM2 (at5g50950), repressed in shoots.

## 3. Discussion

The present work analyzed the response of *A. thaliana* to colonization by *G. diazotrophicus* PAL5, emphasizing the main pathways regulated in plant shoots and roots. Our results confirmed the growth promotion of the inoculated plants, although the presence of the bacteria was restricted to the root tissues. Analyses of gene expression by comparative transcriptomics (RNA-seq) revealed the regulation of distinct gene sets between shoots and roots, indicating specific response pathways in each plant organ.

The plant growth promotion induced by *G. diazotrophicus* has been reported in several crops, including monocots such as sugarcane [27], rice [28], sorghum and wheat [29], and dicots, such as tomato [30] and beans [31]. *A. thaliana* plants inoculated with *G. diazotrophicus* PAL5 show increased shoot and root weight, higher whole canopy photosynthesis, lower whole plant transpiration, and increased water-use efficiency [19]. In our assays, inoculation with *G. diazotrophicus* PAL5 also enhanced the weight of roots and shoots and increased total leaf area. Thus, the beneficial effects of *G. diazotrophicus* PAL5 on the growth promotion of *A. thaliana* provide a promising tool for studying the molecular mechanisms involved in the plant–diazotrophic endophyte interaction, in complement to data available from the inoculation of native plant hosts.

Our transcriptomic analysis revealed several changes in the gene expression of plants inoculated with *G. diazotrophicus* PAL5. Approximately 55.1% of all regulated genes were observed exclusively in shoots, 39.9% exclusively in roots, and only 5.0% in both organs. In shoots, a higher proportion of DEGs was repressed, while in roots, a higher proportion of induced DEGs was observed. Although the bacteria colonize only the roots, our findings show that a robust and organ-specific gene expression response is also observed in shoots. Several studies addressing the proteomic and transcriptomic analysis of *A. thaliana* inoculated by root-specific PGPB also observed the regulation of molecular pathways in shoots, indicating the activation of systemic responses [16,32,33,34].

Plant cell walls provide structural support during plant growth and development and protect against biotic and abiotic stresses [35]. Several microorganisms secrete cell-wall-degrading enzymes, and plants counterattack by reinforcing the composition and structure of the cell wall to restrict microbial colonization [36]. In this study, the DEGs involved in cell wall biosynthesis and degradation were preferentially induced in roots, while such pathways were mostly repressed in shoots. At the roots, the induction of DEGs for cellulose and hemicellulose production [37], extensin activity [38], and pectin degradation enzymes [39] suggests that *A. thaliana* reinforces the cell wall at the site of the *G. diazotrophicus* PAL5 establishment. *G. diazotrophicus* can produce hydrolytic enzymes to degrade the plant cell wall [40]. In shoots where the bacterium was not observed, the repression of this pathway may be related to the weakening of the cell wall required by cell expansion and the higher vegetative growth [41].

The plant response to bacteria often involves the mechanisms of production/detoxification of reactive oxygen species (ROS) [42]. ROS are important secondary messengers that mediate downstream plant immune responses [43]. In our analyses, the peroxidase and glutathione S-transferase (GST) genes were differentially regulated by *G. diazotrophicus* PAL5 between shoots and roots. These pathways were suppressed in the roots, where the bacterium was present, but were induced in the shoots. Inoculation of maize with *Azospirillum brasilense* also led to the down-regulation of oxidative stress genes in roots and up-regulation in shoots, suggesting the presence of finely-tuned interacting mechanisms in plant tissues [44]. The negative regulation of plant defense pathways by beneficial bacteria has been described as necessary to allow the bacteria to colonize and survive within the host plant [45,46]. Thus, it can be hypothesized that the induction of such protective routes in the shoots of *A. thaliana* is in accordance with the inability of *G. diazotrophicus* PAL5 to colonize this region of the plant.

Genes associated with secondary metabolite biosynthesis pathways were mostly induced in inoculated plants. Glucosinolates are well-studied plant metabolites with a key role in plant defense against microbial pathogens [47] and insect herbivores [48]. In cruciferous plants, a regulatory role in root colonization was proposed for glucosinolates [49]. The lower glucosinolate concentration of *A. thaliana* roots supported the colonization by the growth-promotion bacterium *Kosakonia radicincitans* [50]. In this study, a higher number of glucosinolate pathway genes were induced in the roots of inoculated plants, suggesting that *A. thaliana* may activate glucosinolate production to modulate colonization by *G. diazotrophicus* PAL5.

In our analysis, genes related to the biosynthesis of the two subclasses of flavonoids, anthocyanins and flavonols, were mainly induced in the shoots of inoculated plants. These compounds can act in plant defense against herbivores, free radical scavenging, and abiotic stress mitigation [51]. Additionally, flavonols participate in the attraction of beneficial microbiota [52]. The inoculation of sugarcane plants with *G. diazotrophicus* led to the expression of essential regulatory genes from flavonol biosynthesis pathways [53]. The accumulation of flavonols has been suggested as a general plant response to inoculation with beneficial bacteria [45,54,55].

Pathogenesis-related (PR) proteins are essential to plant defense against pathogens, including fungi, bacteria, viruses, insects, and herbivores [56]. During the response to infection by pathogens, plants activate specific PR protein groups with different molecular functions, such as PR-2 (a β-1,3-glucanase), PR-3 (a chitinase family), PR-5 (a thaumatin-like), PR-10 (a ribonuclease-like), PR-12 (a defensin), PR-13 (a thionin), and PR-14 (a lipid-transfer protein) for example [57]. Similarly, multiple studies on PGPB have identified the induction of the PR genes in the host plant [16,33,34]. In our study, PR protein genes were mainly down-regulated in both organs, suggesting the ability of *G. diazotrophicus* PAL5 to suppress such a plant defense response as a colonization strategy.

Heat-shock proteins (HSPs) also play an essential regulatory role in the plant defense response of plant cells, particularly in the quality control of intracellular R proteins and plasma membrane-resident pattern recognition receptors (PRRs) [58]. Among HSP families, the roles of HSP90, HSP70, HSP40, and HSP20 in plant immunity are the most well-characterized to date, in particular during bacterial and viral infections [59]. Here, we identified the repression of genes encoding HSP, especially in the roots of inoculated *A. thaliana* plants. Thus, the negative regulation of HSP genes may be another strategy of *G. diazotrophicus* PAL5 to suppress the components of plant defense.

Biological nitrogen fixation (BNF) stands out among the characteristics potentially beneficial for plant growth in *G. diazotrophicus* [60]. This bacterium possesses the nitrogenase enzyme that converts atmospheric elemental dinitrogen into ammonia [8]. Sugarcane varieties can obtain up to 107 kg N ha^−1^ yr^−1^ fixed by PGPB associated with roots [61], which causes an efficient increment in productivity and reduction in N fertilization [60]. Our findings showed that *G. diazotrophicus* PAL5 induces the expression of genes responsible for nitrate and ammonium uptake and assimilation in the roots and shoots of *A. thaliana*. The coordinated regulation of the genes for the metabolism and transport of nitrogen in inoculated plants suggests the action of this bacterium in providing nitrogen inside the plant tissues.

Although the colonization of *G. diazotrophicus* PAL5 did not cause disease symptoms in *A. thaliana*, a plant defense response was triggered. Such a process is well-documented in various studies about the interaction between endophytic diazotrophic bacteria and their host plants [16,45,46]. In monocots, *G. diazotrophicus* colonization induced transcriptional and proteomic changes in different plant defense response pathways [9,12,53,62,63,64,65,66]. Such data agree with our findings, indicating the modulation of the *A. thaliana* defense system during the interaction. The participation of plant defense mechanisms in the interaction of *A. thaliana* and *G. diazotrophicus* PAL5 was also reported by [19].

## 4. Materials and Methods

### 4.1. Biological Material Sources

The *G. diazotrophicus* PAL5 strains “kanamycin-resistant *G. diazotrophicus* (GD-Kan) and DsRed Fluorescent Protein-labeled *G. diazotrophicus* (GD-F)” [19,67] used in the present study were obtained from the culture collection of the Laboratory of Biotechnology at the Universidade Estadual do Norte Fluminense Darcy Ribeiro (UENF, Campos dos Goytacazes, RJ, Brazil). The seeds of *A. thaliana* (Columbia-0) used were kindly provided by Ph.D. Frederick M. Ausubel (Harvard Medical School, Boston, MA, USA).

### 4.2. Preparation of Bacterial Inoculum

The pre-inoculum was obtained from an isolated colony of *G. diazotrophicus* strain PAL5 (GD-Kan and GD-F) and after growth in DYGS broth [68] at 30 °C and 180 rpm^−1^. After growth, a 5 mL aliquot of the bacterial suspension was transferred to DYGS broth under the same conditions described above. After 12 h of growth, the bacterial cells were sedimented by centrifugation (12,000× *g* for 10 min) and resuspended in sterile distilled water at a cell density of 1.0 × 10^8^ CFU mL^−1^. Kanamycin (50 µg mL^−1^) was added to the medium to cultivate the GD-Kan strain. Kanamycin and streptomycin (50 µg mL^−1^) were added to the medium for cultivation of the GD-F strain.

### 4.3. Growth Promotion of A. thaliana Seedlings Inoculated with G. diazotrophicus PAL5

Seeds of *A. thaliana* were disinfected in 95% ethanol for 1 min and then in 2.5% NaOCl solution for 10 min and finally thoroughly rinsed in sterile water six times. After surface disinfestation, seeds were stratified at 4 °C in the dark for 2 days. Seeds were sown on Petri dishes containing one-half-strength (½) Murashige and Skoog (MS) salt media. After 7 days of growth, the seedlings were transferred to Petri dishes containing 20 mL of the bacterial inoculum [19]. As treatments, 20 replicates of seedlings were inoculated with 1.0 × 10^6^ CFU mL^−1^ *G. diazotrophicus* PAL5 strain GD-Kan, with control seedlings remaining uninoculated. After 3 h of inoculation, the seedlings were individually transferred into 50 mL pots filled with commercial substrate West Garden^®^ (West Garden Ltd.a, São Paulo, SP, Brazil) that was autoclaved for 40 min at 121 °C. The seedlings were incubated at 23 °C, 60% relative humidity, and 70 µmol m^−2^ s^−1^ light (12 h photoperiod) for 50 days. The seedlings were irrigated with 25% Hoagland’s solution [69] every two days.

At 50 days post-inoculation (dpi), the plants were collected and separated into biological replicates for anatomical and morphological analysis. The total leaf area was measured using a leaf area meter (LI-3100C, LI-COR, Lincoln, NE, USA), and the fresh and dry weights of shoot and root parts were determined. Fresh weight was measured using a precision scale immediately after collection. The material was oven-dried at 60 °C for 24 h, and dry weight was determined.

Data were analyzed using variance analysis (ANOVA) followed by a Tukey’s test. Data analyses were carried out using GraphPad Prism 8.0 (GraphPad Software, San Diego, CA, USA). In all cases, the differences were considered significant at *p* < 0.05.

### 4.4. Bacterial Growth Estimation

For bacterial growth estimation, *A. thaliana* seedlings non-inoculated and inoculated with kanamycin-resistant strain (GD-Kan) were removed from the plastic pots after 50-day inoculation and washed in tap water before proceeding with the disinfection procedure. Two leaf disks (0.5 cm diameter) and a 1 cm section of the root (3 cm from the root tip) were used from ten plants in each treatment. The leaf samples were dipped into 70% ethanol for 3 min for superficial disinfection, while the roots were dipped into 1% Chloramine-T for 1 min. After that, the samples were washed three times in ultrapure sterile water. Serial dilutions were carried out, and aliquots of 100 µL were plated on solid LGI-P media [70] containing 50 μg mL^−1^ of kanamycin and incubated for four days at 30 °C. After that, the number of single colonies was counted.

### 4.5. Localization of G. diazotrophicus PAL5 in the Roots of A. thaliana

Aiming to assess the localization of *G. diazotrophicus* PAL5 in *A. thaliana* roots, the DsRed Fluorescent Protein-labeled bacteria (GD-F) was used for plant inoculation based on the method previously described. Regions of 3.0 cm in length, taken at an intermediate point of the root (3 cm from the root tip) were collected and washed in ultrapure sterile water. After that, the roots were cut into cross sections and mounted on slides. The samples were examined using a fluorescence microscope (Zeiss Axiovert 200; Carl Zeiss AG, Jena, TH, Germany). DsRed Fluorescent Protein-labeled bacterial cells were excited with green light (568 nm; 640 ± 60 nm emission filter). Five plants per treatment were used in epifluorescence microscopy analysis.

### 4.6. RNA Extraction and Sequencing

Samples of *A. thaliana* leaves and roots, control and inoculated with *G. diazotrophicus* PAL5 (GD-Kan), were macerated in liquid nitrogen for RNA extraction. According to the manufacturer’s instructions, the total RNA of the samples was extracted with the RNeasy Plant Mini Kit (Qiagen, Valencia, CA, USA). The quantity, quality, and integrity of the total RNA were examined using a Nanodrop 2000 spectrophotometer (Thermo Fisher Scientific, Waltham, MA, USA) and an Agilent 2100 Bioanalyzer (Agilent Technologies, Santa Clara, CA, USA). mRNA was purified from total RNA using Oligo (dT)-attached magnetic beads. The isolated mRNA was cut into short fragments under high temperatures. Then, double-stranded cDNA was synthesized using the SuperScript Synthesis Kit (Invitrogen, Waltham, MA, USA) with random hexamer primers (Illumina, San Diego, CA, USA). The cDNA fragments were purified and washed for terminal reparation and poly(A) addition. The short fragments were connected with adapters. The fragments (200 pb) were PCR-amplified for cDNA library construction. After, the cDNA library was sequenced on the Illumina HiSeq™ 2000 platform (Illumina, San Diego, CA, USA) by the LaCTAD Facility of the Universidade Estadual de Campinas (UNICAMP, Campinas, SP, Brazil). Three biological replications from shoot and root tissues were used for RNA-seq experiments. Each biological replicate consisted of tissues pooled from six individual plants.

### 4.7. RNA-Seq Data Processing and Data Analysis

The reads were aligned to the version 10 *A. thaliana* genome of the Arabidopsis Information Resource (TAIR). The transcripts were reconstructed, and their abundances were estimated with Cufflinks [71] and normalized by fragments per kilobase of transcript per million mapped reads (FPKM). Differentially expressed genes (DEGs) were identified using the Cuffdiff [71] at a false discovery rate (FDR) threshold of 0.05. We used a cut-off *p*-value ≤ 0.05 and log2fold-change ≥ 0.37 for up-regulated genes and a cut-off *p* ≤ 0.05 and log2fold-change ≤ −0.37 for down-regulated genes. The Gene Ontology (GO) was carried out using OmicsBox [25]. MapMan software version 3.6.0 (Max Planck Institute of Molecular Plant Biology, Potsdam, BB, Germany) [26] was used for pathway analysis. All charts (Venn diagram, bar charts, and heatmaps) were drawn using Excel 2022 (Microsoft Corporation, Redmond, WA, USA) and Adobe Illustrator CS6 tools (Adobe Systems Incorporated, San Jose, CA, USA).

## 5. Conclusions

**Figure 6:**
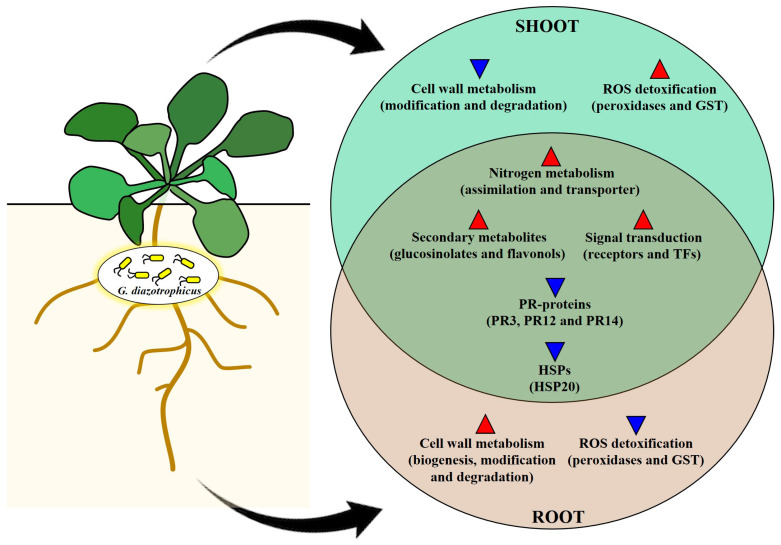
Schematic illustration of the major transcriptional regulation in the shoots and roots of *G. diazotrophicus*-inoculated *A. thaliana*. The overlapping region corresponds to common DEGs in the shoots and roots. The upward red arrows show up-regulated DEGs, and the downward blue arrows show down-regulated DEGs.

*Gluconacetobacter diazotrophicus* PAL5 colonizes and promotes the growth of *A. thaliana.* Our findings confirmed that *G. diazotrophicus* specifically colonizes the root tissues of *A. thaliana* and significantly influences the gene expression of the plant. Figure 6 summarizes the main molecular changes occurring in both the shoots and roots during this association. Some metabolic pathways are similarly activated in the shoot and root tissues of inoculated plants, such as nitrogen metabolism, receptor kinases, transcription factors, and the production of glucosinolates and flavonoids. Also, the down-regulation of genes encoding the pathogenesis-related proteins and heat-shock proteins was observed in both shoot and root tissues. In contrast, pathways involved in cell wall biogenesis and modification and ROS detoxification were differently regulated in shoots and roots. The results of the study contribute to a better understanding of the molecular mechanisms triggered in host plant shoots and roots during interaction with endophytic bacteria, with an emphasis on *G. diazotrophicus* PAL5.

## Figures and Tables

**Table 1:** Overview of the inoculated *A. thaliana* plants RNA sequencing (RNA-Seq) data.

Sample	Raw Reads	Q30%	Reads		Multiple Alignments	
			Mapped	Percentage	Sequenced	Percentage
SC1	37,610,360	94.88	35,427,962	94.20	1,209,120	3.41
SC2	33,674,915	94.52	31,333,299	93.05	1,017,031	3.25
SC3	37,112,472	94.82	34,892,662	94.02	1,067,210	3.06
SI1	33,813,467	94.80	28,484,558	84.24	866,647	3.04
SI2	26,400,110	94.88	24,901,074	94.32	734,767	2.95
SI3	22,157,011	94.99	21,062,186	95.06	664,485	3.15
RC1	19,549,295	90.53	17,201,743	87.99	1,432,636	8.33
RC2	22,796,113	94.20	20,908,142	91.72	1,157,000	5.53
RC3	22,040,564	93.32	19,805,611	89.86	1,533,504	7.74
RI1	29,443,587	92.14	26,091,673	88.62	1,990,799	7.63
RI2	27,813,151	91.92	24,497,311	88.08	1,635,077	6.67
RI3	29,150,837	94.51	26,255,356	90.07	1,624,656	6.19

## Data Availability

The sequencing data were deposited at the NCBI Sequence Read Archive (SRA) under the BioProject accession number PRJNA768887.

## Supplementary Materials

The following supporting information can be downloaded at https://www.mdpi.com/article/10.3390/plants13131719/s1, Table S1: List of differentially expressed genes (DEGs) in the shoots and roots of *Arabidopsis thaliana* inoculated with *Gluconacetobacter diazotrophicus* PAL5 at 50 dpi, Table S2: List of DEGs and their functional classification in terms of Gene Ontology (GO) in *Arabidopsis thaliana* after inoculation by *Gluconacetobacter diazotrophicus* PAL5, Table S3: List of DEGs in different biological roles in *Arabidopsis thaliana* after inoculation by *Gluconacetobacter diazotrophicus* PAL5, Table S4: Transcription factor (TF) genes in the shoots and roots of *Arabidopsis thaliana* plants inoculated by *Gluconacetobacter diazotrophicus* PAL5 at 50 dpi.
