# Supplementary material for: Fine-Tuning of Arabidopsis thaliana Response to Endophytic Colonization by Gluconacetobacter diazotrophicus PAL5 Revealed by Transcriptomic Analysis
Source: Plants (Basel). 2024 Jun 21;13(13):1719. doi: 10.3390/plants13131719 (PMC11244368; doi:10.3390/plants13131719)
Supplement: Supplementary file 1 [file plants-13-01719-s001.zip › Table S4.pdf]

Supplementary Table S4 Transcription factor (TF) genes in the shoots and roots of *Arabidopsis thaliana* plants inoculated by *Gluconacetobacter diazotrophicus* PAL5 at 50 dpi. A prospective function is indicated and down-regulated genes as shown as negative values.

Locus Identifier	Function	Log_2FC
	**Shoots**	
AP2-EREBP family		
At1g43160	ERF113 (RAP2.6), responsive to ABA, JA, SA, heat, cold, salt, osmotic stress, water deprivation, and wounding; chloroplast organization	-1.51
At1g77640	ERF/AP2 transcription factor	-0.65
bZIP family		
At1g21740	DUF630 family protein, putative (DUF630 and DUF632)	-0.54
At2g42380	bZIP34, pollen development	-0.49
At3g58120	bZIP61, regulation of transcription	-0.60
MYB-family		
At1g66380	MYB114 (RER3), multicellular organism development; regulation of anthocyanin biosynthetic process	0.59
At1g70000	MYB-like transcription factor family protein, responsive to karrikin; regulation of anthocyanin biosynthetic process	0.46
At2g46830	CCA1, responsive to cold and organonitrogen compound; regulation of protein-containing complex assembly and gene expression; circadian rhythm; long-day photoperiodism, flowering	0.69
At2g47190	MYB2 (R2R3), responsive to ABA, salt stress, phosphate starvation, and water deprivation	1.06
At4g39250	RADIALIS-like 1, regulation of transcription	0.89
WRKY-family		
At1g18860	WRKY61, transcription factor group II-b	1.18
At2g44745	WRKY12, transcription factor group II-c	-0.60
At4g01250	WRKY22 (group II-e), defense response; responsive to hypoxia and chitin; leaf senescence	-0.59
At4g01720	WRKY47, transcription factor group II-b	0.60
At5g13080	WRKY75 (group II-c), responsive to nutrient levels and stress	0.97
	**Roots**	
AP2-EREBP family		
At1g12610	DDF1 (DREBA1), responsive to heat, water deprivation, freezing; regulation of cell growth, the timing of the transition from vegetative to reproductive phase and gibberellin biosynthetic process; glucosinolate metabolic process	2.13
At1g19210	ERF017, transcription factor	1.64
At1g74930	ORA47, responsive to wounding	0.76
At2g20880	ERF053, responsive to heat, salt stress, and water deprivation	0.96
At3g60490	ERF035, transcription factor	0.42
At4g25490	DREB1B, responsive to cold	0.95
At5g51990	DREB1D, ABA-activated signaling pathway; glucosinolate metabolic process	1.06
DOF-family		
At5g39660	CDF2, flower development; regulation of timing of the transition from vegetative to reproductive phase	0.46

Continued on next page

Table S4 continued		
At5g60850	OBP4, transcription factor	0.52
MYB-family		
At1g09540	MYB61 (R2R3), regulation of stomatal movement; root development	-0.50
At2g47190	MYB2 (R2R3), responsive to ABA, salt stress, phosphate starvation, and water deprivation	-1.04
At3g06490	MYB108 (R2R3), responsive to fungus	-0.73
At4g34990	MYB32 (R2R3), cell differentiation	-0.44
At4g38620	MYB4 (R2R3), responsive to UV-B; negative regulation of sinapate ester biosynthetic process	-0.50
WRKY-family		
At1g18860	WRKY61, factor transcription group II-b	0.56
At2g46400	WRKY46 (group III), defense response; responsive to the bacterium, chitin, SA, water deprivation and hypoxia; regulation of brassinosteroids and jasmonic acid-mediated signaling pathway; lateral root development	0.66
At4g01250	WRKY22 (Group II-e), defense response; responsive to hypoxia and chitin; leaf senescence	-0.63
At4g04450	WRKY42, factor transcription group II-b	0.74
At5g24110	WRKY30 (group III), responsive to ozone, SA and hydrogen peroxide; leaf senescence	-1.70
